# Entomological surveillance for bluetongue virus in Poland: late-season activity and abundance of *Culicoides* vectors in 2024

**DOI:** 10.2478/jvetres-2025-0066

**Published:** 2025-11-26

**Authors:** Małgorzata Kwaśnik, Anna Orłowska, Wojciech Rożek, Magdalena Larska, Jerzy Rola

**Affiliations:** Department of Virology and Viral Animal Diseases, National Veterinary Research Institute, 24-100 Puławy, Poland

**Keywords:** BTV, *Culicoides*, orbivirus, UV light trap, vector competence

## Abstract

**Introduction:**

This study focused on the seasonal activity and species diversity of *Culicoides* biting midges collected from 11 locations along the western border of Poland and major livestock transit routes, and included screening for bluetongue virus (BTV) RNA.

**Material and Methods:**

The sampling was conducted between September and November 2024. Collected biting midges were counted and identified to the species level. The gonotrophic forms of the females were also determined. Pools of insects were tested for the presence of BTV genetic material using a reverse-transcription quantitative real-time PCR.

**Results:**

A total of 13,022 individuals were identified. The results revealed spatial and temporal variation in midge abundance, likely influenced by local environmental conditions. A sharp decline in activity was observed after week 44, coinciding with decreasing ambient temperatures The widespread presence of *Culicoides obsoletus / scoticus* complex, recognised vectors of BTV, was confirmed along with high abundance of *C. punctatus*, a species considered a potential vector. All gonotrophic forms were identified, and 57.3% of females had taken a blood meal, indicating active reproduction and frequent host-animal contact throughout the sampling period. Pools of blood-fed, parous and gravid females were tested for BTV RNA, but all samples returned negative results.

**Conclusion:**

Although no evidence of active BTV circulation was found, the presence of competent vectors and favourable autumn conditions highlights the potential risk of transmission. These findings underscore the need for continued entomological and virological surveillance to support early detection and control of BTV.

## Introduction

A Bluetongue (BT) is an infectious, non-contagious disease of domestic and wild ruminants caused by the bluetongue virus (BTV), a vector-borne orbivirus in the family *Reoviridae* (34). The disease is notifiable to the World Organisation for Animal Health (WOAH) because of its significant economic impact on the livestock sector, which is estimated to be approximately 3 billion USD annually around the world (27). Transmission occurs exclusively *via* haematophagous biting midges of the *Culicoides* genus. Of roughly 1,400 known species, only around 30 are recognised as BTV vectors (23). Vector competence and serotype transmission are region-specific, which elevates the importance of local entomological surveillance. In Central Europe, midges from the Obsoletus and Pulicaris ensembles are the principal vectors (3).

Before 2006, BT was reported only sporadically in Europe, primarily in Mediterranean regions. This changed with the emergence of BTV-8 in Western Europe in August 2006, which led to over 2,000 farms being affected by the end of that year in the Netherlands, Belgium, Germany, France and Luxembourg (41). In the following years, several additional BTV serotypes were detected across Europe. Serotype 1 was reported in Spain, France, Romania and Bulgaria (6), BTV-14 was identified in Russia and Poland (14, 26) and BTV-4 was found in Greece (13), Hungary (12), several Balkan countries (35), Italy (20), France (29, 30), Portugal (1) and Spain (4). Bluetongue viruses 9 and 16 occurred primarily in the Balkans (19), with occasional cases in Western Europe (28). In addition, BTV-8 re-emerged in France and subsequently spread northward and eastward to western Germany (9), Switzerland (10) and Belgium (7, 15, 28).

In 2023, a new epizootic wave of BTV-3 infections emerged, with the first clinical cases reported in the Netherlands in September on four sheep farms. By mid-December, the number of confirmed outbreaks had increased dramatically, reaching 5,884 (11). In October 2023, Germany reported its first BTV-3 case in the Kleve district, located near the Dutch border (39). In 2024, the virus spread rapidly across Europe, with confirmed cases in France, Luxembourg, Austria, Italy, Switzerland, Denmark, Portugal, Spain and the United Kingdom (37). This serotype caused severe clinical disease in sheep and higher mortality rates than those observed during the BTV-8 epidemic of 2006–2008. The virus also had a significant impact on cattle, affecting commercial dairy herds, suckler cows and beef cattle, and mixed-use cows on small farms. Clinical consequences included a sharp increase in abortions and premature births, severe clinical signs with mortality reaching up to 20%, and prolonged losses in milk production (33, 38).

Poland, officially free of BT since 2010, has become increasingly at risk because of the rapid eastward spread of BTV-3 from the western countries of Europe. Unlike BTV-8, BTV-3 has spread rapidly across Europe, covering approximately 25 km per week, which might be the outcome of changes in vector dynamics or climate conditions (2). This rapid spread was most likely facilitated by favourable weather conditions for midge activity and the virus’ ability to overwinter in certain regions. In response to the elevated risk of BTV-3 introduction – both due to its rapid spread across Europe and the potential movement of infected animals into Poland – the national authorities have implemented a targeted surveillance programme. This programme focuses on *Culicoides* populations along Poland’s western border and major livestock transit routes. The objectives of this study were to assess the abundance and species composition of local *Culicoides* populations, to note vector species and their gonotrophic status as indicators of transmission potential in particular, and to test midges for the presence of BTV.

## Material and Methods

### *Culicoides* collection in the hotspots

**Fig. 1.**
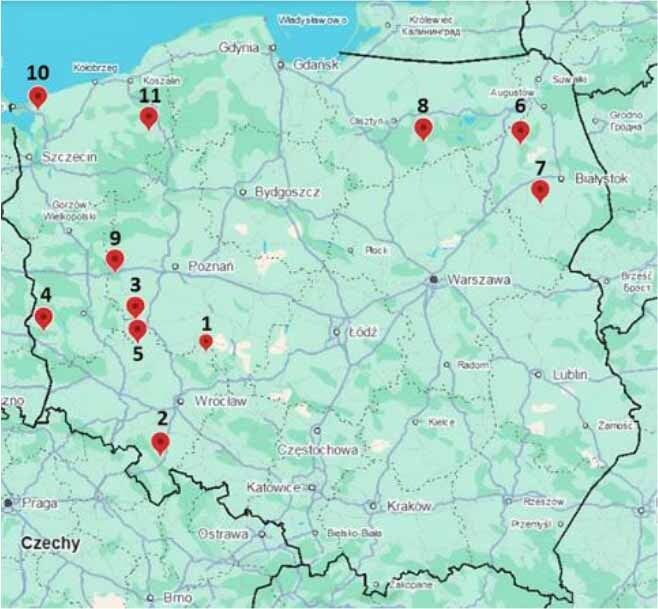
Map of 11 *Culicoides* sampling locations in Poland generated using Google Earth 10.81.0.1. The numbers correspond to those in Table 1

Onderstepoort-type traps for biting midges were installed at 11 locations in seven Polish voivodeships (Fig. 1) and were operated between September and November 2024. The coordinates of the trapping locations are provided in Table 1. The following criteria were used to select the trapping sites: proximity to the western border and to main animal transport routes, density of reservoir species (cattle and sheep), and an assessed risk of virus incursion from neighbouring countries. Midges were primarily caught in locations strategic for animal movements within the country between EU Member States and third countries, *i.e*. control points where transport is interrupted for a stopover of more than 24 h and places where animals are gathered, transhipped or purchased for the purpose of forming consignments for trade. The traps were placed in a location sheltered from rain and wind and without competing light sources near the trap, and were hung so that the UV light bulb was 1.5–2 m above the ground. The trap container was approximately three-quarters full of water with a few drops of detergent to reduce surface tension and cause the insects to sink to the bottom of the tank. The traps were turned on one hour before sunset and turned off the next morning once a week in the period from 9 September to 29 November 2024. The day after collection, the insects were drained, suspended in 70% ethanol and stored at room temperature until processing.

**Table 1.** Summary of *Culicoides* midge collections by Polish county, including collection dates, total number of collected and proportion of blood-fed or post-blood-feeding individuals

No.	County	Coordinates	Voivodeship	Collection start	Collection end	Total midges	% Blood-fed / Post-blood-feeding
1	Milicki	51.55828 17.42570	Dolnośląskie	10/09/2024	25/11/2024	1,874	75.7
2	Ząbkowicki	50.54168 16.73937	Dolnośląskie	09/09/2024	28/10/2024	719	65.8
3	Wschowski	51.84626 16.32727	Lubuskie	17/09/2024	28/10/2024	120	22.2
4	Żarski	51.71833 14.90194	Lubuskie	11/09/2024	25/11/2024	1,467	39.8
5	Sieradzki	51.62588 16.36884	Łódzkie	11/09/2024	29/10/2024	311	50.4
6	Augustowski	53.45298 22.54266	Podlaskie	13/09/2024	12/11/2024	1,636	47.7
7	Białostocki	52.86790 22.79496	Podlaskie	13/09/2024	22/11/2024	176	90.9
8	Szczycieński	53.52915 20.95410	Warmińsko-Mazurskie	16/09/2024	25/11/2024	4,480	67.2
9	Nowotomyski	52.29694 15.99486	Wielkopolskie	10/092024	21/11/2024	920	69.5
10	Kamieński	53.84892 14.66862	Zachodnio-Pomorskie	12/09/2024	27/10/2024	707	37.0
11	Szczecinecki	53.67671 16.49079	Zachodnio-Pomorskie	12/09/2024	29/11/2024	606	64.4

### Morphological analysis of midges

Biting midges were counted and identified to the species level based on the identification key of Mathieu *et al*. (21) using a stereoscopic microscope. The gonotrophic forms of the female midges were also determined based on abdominal traits observed under an SDF PLAPO 1XPF objective in an SZX16 microscope (Olympus, Tokyo, Japan). Pools consisting of 20 individuals of the same insect species originating from the same trapping session were prepared for the detection of BTV RNA.

### BTV testing

Midges of the same species were pooled as blood-fed and parous specimens together and BTV RNA was screened for in these pools. A low number (n = 9) of pools containing nulliparous, blood-fed, gravid and parous *Culicoides* together or nulliparous and parous gonotrophic forms together were subjected to BTV testing. Specimens were homogenised in phosphate-buffered saline using mechanical disruption with 1.4 mm ceramic (zirconium silicate) beads (Lysing Matrix D; MP Biomedicals, Irvine, CA, USA) in a TissueLyser instrument (Qiagen, Hilden, Germany). The homogenisation protocol consisted of two 45-s cycles at 6,500 rpm, with samples cooled on ice between cycles. Total RNA was extracted from 200 μL of homogenate using the IndiMag Pathogen Kit (Indical Bioscience, Leipzig, Germany) on an IndiMag 48 automated extraction system (Indical Bioscience), following the manufacturer’s protocol. Extracted nucleic acids were either used immediately in a real-time reverse-transcription PCR (RT-PCR) or stored at –80°C until further analysis. A protocol targeting a fragment of the *NS3* gene segment was employed to detect BTV RNA, following the standard operating procedures of the European Union Reference Laboratory for Bluetongue.

Briefly, 2 μL of RNA were mixed with 1 μL of each primer (10 μM) for BTV and 18S rRNA and denatured at 95°C for 5 min, then immediately cooled on ice for 3 min. Next, a mixture containing the BTV probe (0.2 μM), the *Culicoides* 18S rRNA probe (0.2 μM), 12.5 μL of 2× RT-PCR buffer, and enzyme mix was made to give a final reaction volume of 25 μL. A real-time RT-PCR was performed in a QuantStudio 6 system (Applied Biosystems/ThermoFisher Scientific, Singapore) following a programme of reverse transcription at 45°C for 10 min and 1 cycle at 95°C for 10 min, followed by 40 cycles at 95°C for 15 s and 60°C for 1 min. Samples were classified as positive if the threshold cycle (Ct) value was lower than 40.

## Results

**Fig 2.**
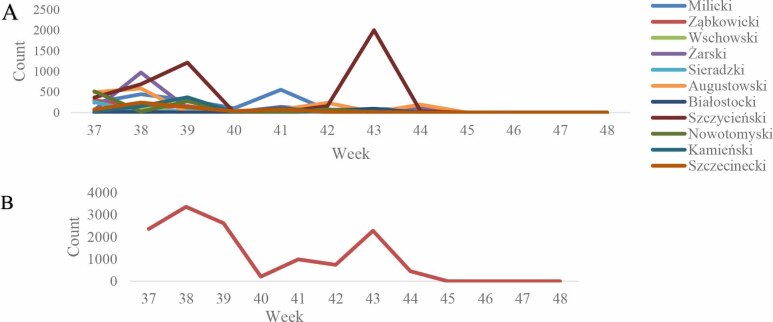
A - Temporal dynamics of *Culicoides* abundance across 11 trapping sites in Poland. Individual trapping locations are differentiated by colour; B - Graph of the dynamic of *Culicoides* numbers trappe

A total of 13,022 individual *Culicoides* females were identified in the 11 locations selected for the entomological monitoring. The number of midges collected per site ranged from 120 to 4,480. In most locations, trapping was carried out once a week from the beginning of September until the end of November. The highest activity of *Culicoides* was observed in the Szczycieński district (No. 8), and was characterised by two intense peaks of abundance. The Milicki (No. 1), Żarski (No. 4) and Augustowski (No. 6) districts also exhibited noticeable surges in *Culicoides* counts, although these were shorter in duration and less pronounced than in Szczycieński. In the remaining locations, midge abundance remained low and relatively stable throughout the observation period. Notably, midge activity across all sites ceased almost entirely after week 44, indicating a sharp seasonal decline. The dynamics of *Culicoides* abundance across the 11 trapping sites is presented in Fig. 2.

**Fig. 3.**
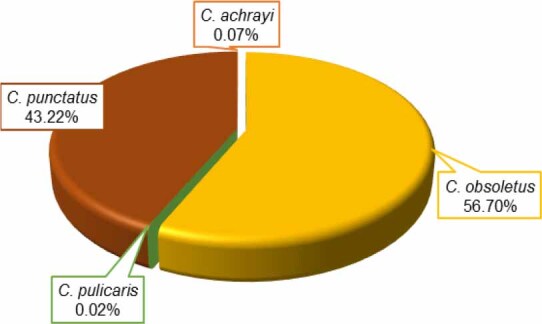
Pie chart showing the species composition of *Culicoides* specimens collected during trapping conducted from 9 September to 29 November at 11 sites in Poland

Regardless of the trapping site, the most abundant species was *C. obsoletus / scoticus* complex (7,383 individuals; 56.7%). *Culicoides punctatus* made up almost all of the remainder (5,628 individuals; 43.2%). Occasionally, *C. achrayi* and *C. pulicaris* were also recorded (Fig. 3).

**Fig. 4.**
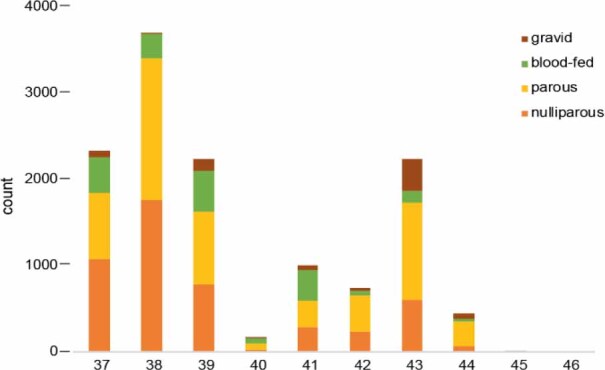
Gonotrophic structure of *Culicoides* populations captured weekly in 2024, showing the number of nulliparous, parous, blood-fed and gravid females

Midges were collected in all gonotrophic stages (Fig. 4): nulliparous (females prior to their first blood feeding), parous (females that have digested a blood meal and laid eggs), blood-fed (females with blood-engorged abdomens) and gravid (females carrying eggs). A total of 8,006 females exhibited evidence of prior blood feeding (classified as blood fed, parous or gravid), with blood-fed females comprising 14% of the total sample. The bar chart presents the weekly distribution of *Culicoides* females by gonotrophic stage from weeks 37 to 46. Two major peaks in abundance are evident – in weeks 38 and 43 – dominated by parous and nulliparous females, reflecting high reproductive activity. Gravid and blood-fed individuals appear in moderate numbers, with gravid forms peaking in week 43. After week 44, total counts decline rapidly, and by week 45, midge activity has nearly ceased, marking the end of the reproductive season.

Details of the trapping locations including their coordinates, durations of the sampling periods, total specimen counts and the proportion of blood-fed or post-blood-feeding individuals are presented in Table 1.

A total of 94 *Culicoides* pools, comprising 2,742 mainly blood-fed and parous (post-blood-feeding) specimens from the *C. obsoletus / scoticus* complex and *C. punctatus*, were tested for the presence of BTV RNA. Bluetongue virus RNA was not detected in any of the tested pools, whereas 18S rRNA of *Culicoides* was present in all samples.

## Discussion

The results provide insight into the seasonal dynamics and species composition of *Culicoides* biting midges collected at 11 locations in Poland selected as hotspots for BTV introduction. The sites were identified as locations where the risk was high of arbovirus emergence or transmission *via* stepwise introduction of infected animals, rather than the risk being high of the spread of disease associated with the migration of the BTV vector and the wild reservoir (17, 18, 31). The targeted monitoring was performed from September to November 2024 and was prompted by the increasing risk of BTV-3 in Poland. The study was designed to answer two questions. Firstly, whether the activity and abundance of midges in selected locations would allow BTV to be maintained and transmitted if infected ruminants were introduced. Secondly, whether *Culicoides* surveillance could be used for early warning in the event of BTV emergence. We were able to demonstrate that the temporal and spatial variation in midge abundance reflected the influence of regional environmental conditions and aligned with previously reported patterns in Central and Northern Europe (5, 22). Therefore, the activity of BTV vectors at the hotspots was probably sufficient for further transmission of the virus. This also points to the need to introduce additional biosecurity measures in the locations which are strategically important for BTV emergence prevention. The measures should extend to the use of repellents, limiting the creation of vector breeding sites such as damp or wet decomposing vegetation, manure and dung as well as avoiding places in closest proximity to water reservoirs (40).

This need for targeted biosecurity is particularly relevant given that the high vector habitat suitability associated with water-rich environments was reflected in the entomological findings. Three of the top four sites by midge catch have high water coverage. The Szczycieński district exhibited the highest midge activity, characterised by two abundance peaks during weeks 38 and 43 of the year. These fluctuations likely corresponded to times of favourable temperature and humidity conditions, which may influence both the emergence and reproductive cycles of *Culicoides* (24). We described the first BTV-3 case in a European bison in the same region, and subsequently viral RNA was detected in the midges from the same place (16). We were thus able to confirm BTV infection in the vector, but this was only achieved after the animal’s death and indicated further circulation of the virus in the environment. Using insects, we were unable to predict any impending infection. Nevertheless, we can see the potential in entomological surveillance, not only for early detection but also as a non-invasive means of monitoring BTV spread. The low midge numbers in the Wschowski and Białostocki counties suggested either less suitable breeding habitats or the influence of local microclimatic conditions suppressing population growth. Therefore, these places had lower utility as hotspots for targeted BTV surveillance. A notable seasonal pattern was the abrupt decline in midge activity after week 44 across all sites, coinciding with decreasing ambient temperatures. This finding supports the dependence of *Culicoides* activity on temperature, with reproduction and host-seeking decreasing when temperatures drop below the levels needed for their development (36).

Over 99% of *Culicoides* belonged to the *C. obsoletus / scoticus* complex or were *C. punctatus*, in nearly equal proportions. Only single individuals of *C. achrayi* and *C. pulicaris* were identified. The *C. obsoletetus / scoticus* complex is a taxonomic group of nearly indistinguishable species. Accurate identification often requires molecular tools because of their morphological similarity. This species composition of the total catch was consistent with patterns observed in other European countries, where members of the *C. obsoletus / scoticus* complex and *C. punctatus* were also reported as predominant, especially in temperate zones during the autumn months (25). The *C. obsoletus / scoticus* complex is known as the main vector of BTV in temperate Europe (8), while the contribution of *C. punctatus* to BTV transmission remains unconfirmed.

The presence of all female gonotrophic forms – nulliparous, parous, blood-fed, and gravid – indicates active reproductive cycling and host contact throughout the sampling period. The peaks in parous and nulliparous females during the weeks of highest abundance suggest intense transmission potential during those intervals. The high proportion of females that had already taken a blood meal indicates active reproduction within the *Culicoides* population during the investigated period. This suggests that environmental conditions such as temperature, humidity, and host availability were favourable to intensive population growth (32). In our study, particular attention was given to parous, blood-fed and gravid individuals, which together accounted for 57.3% of the specimens collected. Pools composed of these midges that had been in contact with animal blood, grouped by species, were analysed for the presence of BTV RNA. All tested samples yielded negative results. The absence of BTV genetic material in the examined *Culicoides* specimens may indicate a lack of active virus circulation within the vector population at the time of sampling. This suggests that, despite favourable environmental conditions, the vector was not infected during the study period. However, the presence of blood in more than half of the females indicated recent host contact, implying that virus transmission could have occurred if BTV had been present in the host population. *Culicoides* trapping in this study was conducted in the period prior to the detection of BTV-3 infection in cattle in Poland, which occurred in late November 2024; therefore, the chance of detecting BTV-3 RNA in a putative insect carrier was low. At the end of October 2024, BTV-3 RNA was detected in Poland’s Wolin National Park in the Zachodnio-Pomorskie voivodeship in one pool of *C. punctatus* caught two weeks after a BTV-3 infection had been confirmed in a European bison inhabiting the park’s bison game reserve. However, the Ct of 32.8 indicated an ongoing transmission cycle, albeit with low virus circulation as only one insect pool tested positive and only one bison was BTV-3 positive on the affected bison reserve (16). Low circulation of BTV-3 in the environment and the presence of its putative vector was also reported in Germany in a study to detect viral RNA in *Culicoides* pools caught near the border with the Netherlands (BTV-3 distribution areas) from 26 September to 9 November 2023 (39), the area and time corresponding to those of the detection of BTV-3 on a German sheep farm.

Circulation of BTV-3 in Poland is likely to continue and intensify, especially in the season of biting midge activity; therefore, entomological and virological surveillance should be continued, particularly during periods of increased transmission risk.

## Conclusion

The results of this study identify early autumn as the peak period of *Culicoides* activity, emphasising the importance of continued entomological surveillance during this time due to favourable conditions for virus transmission. The findings also highlight the necessity of implementing region-specific monitoring strategies to enhance the prediction and mitigation of BTV introduction and spread in Poland. Our surveillance confirmed the widespread occurrence of the *C. obsoletus / scoticus* complex, a known BTV vector, and a similarly high abundance of *C. punctatus*, which may also play a role in transmission. Notably, *C. imicola* – the primary vector of BTV in Africa and southern Europe (24) – was not detected in the study areas. No BTV genetic material was identified in any of the analysed *Culicoides* pools, indicating no evidence of active virus circulation at the time of sampling.
